# Neutrophil heterogeneity in health and disease: a revitalized avenue in inflammation and immunity

**DOI:** 10.1098/rsob.120134

**Published:** 2012-11

**Authors:** Martina Beyrau, Jennifer Victoria Bodkin, Sussan Nourshargh

**Affiliations:** William Harvey Research Institute, Barts and The London School of Medicine and Dentistry, Queen Mary University of London, Charterhouse Square, London EC1M 6BQ, UK

**Keywords:** neutrophil, neutrophil subset, neutrophil phenotype, inflammation, neutrophil plasticity

## Abstract

Leucocytes form the principal cellular components of immunity and inflammation, existing as multiple subsets defined by distinct phenotypic and functional profiles. To date, this has most notably been documented for lymphocytes and monocytes. In contrast, as neutrophils are traditionally considered, to be short-lived, terminally differentiated cells that do not re-circulate, the potential existence of distinct neutrophil subsets with functional and phenotypic heterogeneity has not been widely considered or explored. A growing body of evidence is now challenging this scenario, and there is significant evidence for the existence of different neutrophil subsets under both physiological and pathological conditions. This review will summarize the key findings that have triggered a renewed interest in neutrophil phenotypic changes, both in terms of functional implications and consequences within disease models. Special emphasis will be placed on the potential pro- and anti-inflammatory roles of neutrophil subsets, as indicated by the recent works in models of ischaemia–reperfusion injury, trauma, cancer and sepsis.

## 2. Emergence of leucocyte subsets

Paul Ehrlich published his landmark paper describing a technique to stain blood films and perform differential blood counts in 1879 [1]. Until about 50 years ago, this approach was routinely used to analyse leucocytes as distinct and uniform subsets of immune cells, largely defined by their morphology, uptake of specific indicators and/or expression of specific antigens. The modern era of cell biology has unravelled the complexity of the host's defence system, not only through the diversity of its molecular pathways, but also through the heterogeneity of the cellular components. For example, lymphocytes, the principal components of adaptive immunity, again originally described based on their appearance in peripheral blood smears, are now known to be formed by many functionally distinct subsets [2–5]. Similarly, monocytes (or mononuclear phagocytes), a cell lineage with a critical role in the innate immune response to pathogens, as well as wound healing, are well accepted to be morphologically, phenotypically and functionally heterogeneous [6]. Neutrophils, however, the host's first line of defence against invading pathogens, have long been considered to be a relatively homogeneous cell population. A major contributing factor to this misconception is that, conventionally, neutrophils have been considered as short-lived ‘kamikaze’ cells that arrive rapidly at sites of infection and injury, are over-exuberant in their activity, and die within the infiltrated tissue. A growing body of evidence has challenged this dogma, suggesting that neutrophils may have a longer lifespan than previously considered (e.g. 5.4 days in humans) [7], can be detected in lymphoid organs [8], are associated with the pathogenesis of numerous chronic inflammatory disorders and exhibit elaborate interactions with components of the adaptive immune response [9]. In line with the above indications of a broader role for neutrophils in immunity and inflammation than is conventionally considered, emerging evidence also indicated the presence of different neutrophil subsets with distinct phenotypic and functional profiles in various disease scenarios [10–14]. Furthermore, contrary to being ‘dead-end’ cells, neutrophils can exhibit reverse transmigration and re-enter the circulation [15,16], a phenomenon that has also been associated with a shift in neutrophil phenotype towards a pro-inflammatory state [16,17] and a resultant impact on dissemination of systemic inflammation [16].

Collectively, while neutrophil biologists have been aware of neutrophil functional heterogeneity for many years [18], recent evidence has cemented the concept of distinct neutrophil subsets based on defined molecular markers. This review aims to discuss the key concepts in this emerging field.

## 3. Neutrophil phenotype change and emerging subsets

As long ago as 1920, it was reported that neutrophils are not the homogeneous leucocyte subset typically envisaged, and that circulating neutrophils can show significant differences in parameters, such as phagocytosis, protein synthesis and oxidative metabolism [18]. Until recently, this concept attracted little mechanistic attention, with only a limited number of studies addressing the potential functional consequences and the underlying molecular basis of neutrophil phenotypic differences. Emerging data have now confirmed that rather than being an end-stage uniform cell population, neutrophils can show a great level of plasticity and develop distinct phenotypes and/or subsets in response to a wide range of physiological (e.g. impact of age) and pathological (e.g. inflammation and infection) conditions, as detailed below. A contributing factor to the revitalized surge in this field has been the availability of new and highly specific markers for neutrophils, such as antibodies directed against the mouse neutrophil-specific antigen Ly-6G [19].

There is now evidence for the existence of different neutrophil subsets, identified through expression of specific molecular markers in healthy individuals, and although details of their functional consequences have yet to be fully elucidated, a number of exciting avenues are emerging (table 1). Two specific examples are given below.

**Table 1.** Molecules that characterize physiologically occurring neutrophil subsets. Shown are key molecules that are differentially expressed on subsets of neutrophils under physiological conditions and their possible functions. BM, bone marrow; GI, gastrointestinal; GPI, glycosyl-phosphatidylinositol; ICAM-1, intercellular adhesion molecule-1; Ig, immunoglobulin; PECAM-1, platelet endothelial cell adhesion molecule-1; RA, rheumatoid arthritis; rTEM, reverse transendothelial cell migration.

protein	characteristics	expression in neutrophils	function in neutrophils
OLFM4	— glycoprotein — expressed in myeloid cells and the GI tract	— specific granules of 20–25% of human circulating neutrophils	— inhibits intracellular killing of bacteria via inhibition of cathepsin C
NB1 (CD177)	— GPI-anchored cell surface receptor — adhesion molecule	— about 30–70% of human circulating neutrophils	— binds with high-affinity to PECAM-1 — associates with the protease PR3 and may facilitate its cell surface localization — implicated in transendothelial cell migration
ICAM-1 (CD54)	— cell surface glycoprotein — adhesion molecule — Ig superfamily member — expressed in leucocytes and endothelial cells	— very low on circulating neutrophils — on rTEM neutrophils *in vitro* and on 1–2% of circulating neutrophils in chronic inflammation (e.g. RA) — on a small subset of neutrophils post local I-R injury *in vivo*	— binds integrins αLβ2 and αMβ2 — ICAM-1^high^ neutrophils associated with dissemination of systemic inflammation — endothelial ICAM-1 aids transendothelial cell migration
CXCR4 (CD184)	— chemokine receptor	— upregulated on senescent neutrophils	— upregulation aids sequestration of aged neutrophils back to the bone marrow
CXCR2 (CD182)	— chemokine receptor	— downregulated on senescent and rTEM neutrophils, and on neutrophils from aged mice	— downregulation impairs neutrophil migration into inflammatory sites

### 3.1. Olfactomedin 4

Olfactomedin 4 (OLFM4) is a glycoprotein that has been suggested to act as a tumour suppressor [20] and has recently been identified in specific granules of approximately 25 per cent of circulating human neutrophils [21], where it inhibits the activation of several granular proteases, including cathepsin C, neutrophil elastase, cathepsin G and proteinase 3 (PR3). This indicates that expression of OLFM4 could negatively regulate the efficiency of bacterial killing in a subset of neutrophils [21,22].

### 3.2. CD117

Another molecule whose expression defines a subset of neutrophils is the surface glycoprotein CD177 (NB1), a 55 kDa glycosyl-phosphatidylinositol-anchored receptor that is expressed at varying levels on circulating neutrophils in healthy human subjects [23,24]. Several distinct functions have recently been attributed to CD177, including high-affinity binding to the adhesion molecule platelet endothelial cell adhesion molecule-1 (PECAM-1) and the ability of the molecule to associate with the serine protease PR3 [25,26]. The functional implications of CD177 interactions with PECAM-1 and PR3 have recently been addressed in the context of neutrophil transmigration. Briefly, in addition to the established role of PECAM-1 in neutrophil transmigration [27], there is now direct evidence for the involvement of CD177 and PR3 in neutrophil migration through cytokine-stimulated cultured endothelial cells [26]. As CD177 expression on neutrophils is increased in patients with severe bacterial infections, it is potentially possible that under certain inflammatory conditions, CD177-positive neutrophil subsets may support increased neutrophil tissue infiltration as aided by the associated cell surface PR3. However, while the proportion of CD177-expressing neutrophils are increased in the autoimmune disorder Wegener's granulomatosis, a condition strongly associated with the presence of anti-neutrophil cytoplasmic antibodies directed against PR3, CD177-negative neutrophils can also express membrane-associated PR3 [24].

Collectively, as there is evidence for pathogenic ability of neutrophil subsets identified in healthy subjects, there is now a need for better understanding of the functional implications of these subsets under both physiological and disease conditions. Furthermore, as well as the existence of neutrophil subsets with distinct phenotypes under homeostatic conditions, neutrophil phenotypes can also be regulated by physiological responses and phenomena, as illustrated below.

### 3.3. Impact of transmigration on neutrophil phenotype

**Figure 1.**
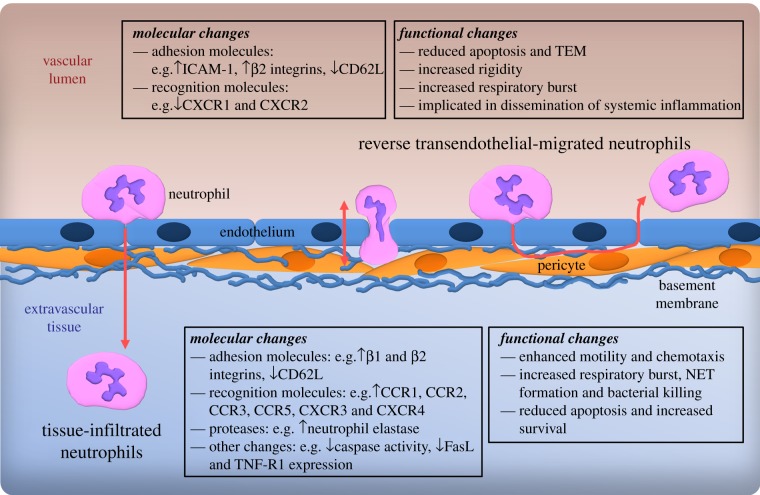
Changes in neutrophil phenotype post-TEM. Summarized are molecular and functional changes reported in conjunction with neutrophil TEM/tissue infiltration and neutrophil reverse transendothelial migration through endothelial cells. The latter may involve movement of neutrophils within the transmigration pore in an abluminal-to-luminal direction or, under extreme conditions, migration from the subendothelial space back into the vascular lumen. FasL, Fas ligand; NET, neutrophil extracellular trap; TEM, transendothelial cell migration; TNF-R1, tumour necrosis factor receptor type 1.

Neutrophil transmigration out of blood vessels involves close contact with the different components of venular walls, namely endothelial cells, venular basement membrane and pericytes [27]. It is well accepted that neutrophil interaction with these structures induces a number of functional and phenotypic changes in neutrophils that aid their continued migration through the venular wall, as well as their migration and effector responses in the extravascular tissue (figure 1) [28]. Key changes include altered expression of adhesion molecules (e.g. increased expression of the integrins α2β1, α4β1 and α6β1), recognition molecules (e.g. increased expression of chemokine receptors CCR1, CCR2, CCR3, CCR5, CXCR3 and CXCR4) and proteases (e.g. increased expression of neutrophil elastase) [28,29]. Furthermore, transendothelial cell-migrated and tissue-infiltrated neutrophils exhibit altered functions such as an extended half-life when compared with their circulating counterparts [30,31], increased migratory capacity, increased cytotoxicity related to increased respiratory burst or neutrophil extracellular trap (NET) formation, and protease release [29,32].

While normal neutrophil transmigration can alter their phenotype in a manner that can support a physiologically efficient immune response, it now appears that disrupted dynamics of neutrophil transendothelial cell migration (TEM) may generate pathologically relevant neutrophil subsets. Typically, in response to an inflammatory signal, neutrophils are recruited to sites of infection, inflammation or injury from the vascular lumen in a luminal-to-abluminal fashion. Once in the tissue, they are classically considered to be phagocytosed by tissue macrophages after fulfilling their effector functions and dying in a programmed manner through apoptosis. A number of studies have shown, however, that as previously reported for monocytes [33,34], neutrophils have the ability to undergo reverse TEM (rTEM), a term that we have used here to describe the movement of neutrophils in an abluminal-to-luminal direction within the transmigration pore or, under extreme conditions, from the subendothelial space back into the vascular lumen [15–17]. Intriguingly, Buckley *et al*. [17], who first reported on the phenomenon of neutrophil rTEM using human neutrophils within an *in vitro* flow model, also noted a distinct change in the phenotype of rTEM neutrophils when compared with blood- and normal-transmigrated (i.e. luminal-to-abluminal) human neutrophils. Reverse TEM of neutrophils through tumour necrosis factor (TNF)-stimulated cultured endothelial cells led to the formation of a neutrophil subset characterized by high expression of intercellular adhesion molecule-1 (ICAM-1) and low expression of the chemokine receptor CXCR1 (ICAM-1^high^CXCR1^low^) [17] (figure 1). This subset of human neutrophils also showed greater rigidity, lower tendency to transmigrate through endothelial junctions again and an increased ability to generate reactive oxygen species (ROS), and was less prone to apoptotic cell death when compared with freshly isolated neutrophils [17]. Interestingly, small but significantly elevated levels of neutrophils with an ICAM-1^high^CXCR1^low^ phenotype were detected in patients with the chronic inflammatory disorders rheumatoid arthritis and atherosclerosis, suggesting a potentially pathogenic role for this subset of cells. Further evidence supporting a detrimental role for rTEM neutrophils was provided by work conducted in our laboratory, presenting the first direct evidence for the occurrence of rTEM in a mammalian system [16]. In this study, using confocal intravital microscopy to analyse the dynamics of neutrophil transmigration within inflamed mouse cremaster muscles, rTEM was found to be most pronounced following ischaemia–reperfusion injury [16]. Importantly, rTEM correlated with the emergence of a subset of ICAM-1^high^ murine neutrophils (which were also functionally primed for enhanced ROS generation) in the pulmonary microcirculation and development of lung inflammation. Collectively, these findings show that rTEM can lead to the generation of a pathogenic subset of neutrophils, a subset that may be involved in dissemination of systemic inflammation from a primary site of injury to distant organs [16].

### 3.4. Impact of neutrophil ageing (senescence) on neutrophil phenotype

The number of circulating neutrophils is dependent on the balance between liberation of fresh neutrophils from the bone marrow and sequestration of ‘old’ (senescent) neutrophils in peripheral tissues for destruction. Circulating neutrophils are generally considered to be short-lived cells with an approximate circulation time of 6–8 h in humans [35]. Interestingly, a recent *in vivo* labelling study by Pillay *et al.* [7] has indicated a significantly longer circulating neutrophil lifespan of 5.4 days in humans and 90 h in mice. While these findings are of tremendous interest, they deviate from the findings of several previous studies, and the validity of the mathematical modelling used by Pillay and co-workers to calculate neutrophil circulation times has attracted some concerns [36,37]. Of importance, the model is based on the assumption of a homogeneous circulating neutrophil pool, which is a simplification of the physiological scenario where blood neutrophil phenotype is impacted by numerous factors, such as maturation and activation state.

Maturation of circulating neutrophils can be ascertained by numerous parameters, most notably by the development of nuclear segmentation. Specifically, immature neutrophils residing in the bone marrow exhibit a round nuclear morphology that is gradually elongated to form a banded nucleus as neutrophils mature. During full maturation and coinciding with their release into the peripheral circulation, nuclei form their characteristic three- to five-lobed morphology [38]. This phenomenon is clearly illustrated during inflammatory neutrophilia when the release of immature neutrophils into the circulation leads to the presence of an increased proportion of banded neutrophils in blood [38,39]. Release of mature neutrophils from the bone marrow is mediated by several chemokine axes—namely the CXCR4/stromal cell-derived factor-1 (SDF-1), CXCR2/interleukin-8 (IL-8) and the very late antigen-4/vascular cell adhesion molecule-1 axes, which are modulated in response to systemic cues [35,40]. Once in blood, neutrophils can be attracted out of the circulatory systems by locally released inflammatory mediators, such as chemokines, in response to infection, injury or inflammation as part of the host's innate immune response. Neutrophils that remain in the vasculature ‘age’ within the circulation and become senescent, acquiring an altered phenotype with compromised functionality in innate immunity that requires them to be cleared from the blood [41]. Senescent neutrophils actively synthesize and express increased levels of CXCR4, which enhances their sensitivity to SDF-1, promoting their homing to the bone marrow. This process is further supported by a reduction in CXCR2-mediated responses of senescent neutrophils, rendering them less sensitive to chemokines, and so reduces their migration to sites of inflammation. Any remaining neutrophils become apoptotic within the circulation and are cleared by the spleen [35,41–43]. Functional differences have been observed in human neutrophils aged *in vitro*, showing reduced degranulation and respiratory burst compared with freshly isolated neutrophils. Furthermore, a decline in chemotactic, phagocytic and shape-change abilities could be correlated with increasing levels of apoptotic neutrophils [44]. In an elegant study, Suratt *et al.* [45] provided evidence for phenotypic differences between immature bone marrow-, blood- and tissue-infiltrated murine neutrophils *in vivo*. On infusion of radio-labelled cells into recipient mice, the homing site of the cells was governed by the level of maturity and activation state of the cells, with bone marrow cells homing to the marrow, while activated tissue-infiltrated cells homed predominantly to the liver, presumably for degradation. Blood-derived neutrophils showed an intermediate phenotype, partitioning between these two locations. As well as providing evidence for the bone marrow as a site of not only release, but also subsequent retention of neutrophils, the findings of this study demonstrate the divergence in behaviour of circulating neutrophils as determined by the age and activation of the cells.

### 3.5. Neutrophil phenotype in the ageing mammal

Changes in neutrophil phenotype have been reported to occur depending on gender [46,47], season [48] and also throughout the mammalian lifespan. These findings build on a growing body of evidence describing dynamic changes in the immune system occurring throughout the host's life. This includes during foetal and neonatal stages [49], upon the transition from childhood to adolescence [47], and during advancing years. The function of the immune system is profoundly impacted by ageing, resulting in a state of diminished ability to fight and clear pathogens in old age, a physiological state termed ‘immunosenescence’. This state impacts both the adaptive and innate components of the immune system, though much more is known about the former (e.g. a decline in B- and T-cell repertoire and function) [50]. Although the impact of age on cells of the innate immune system has proved hard to define, there are indications of alterations in neutrophil function in advanced age, as discussed below. The reader is referred to other sources for reviews of the impact of ageing on changes in phenotype and functions of macrophages, dendritic cells and natural killer cells [51,52].

In parallel with reduced function of the immune system with increased age, aged mammals show a greater susceptibility to infections and chronic inflammatory conditions [50–52]. Ageing is associated with a mild but chronic systemic inflammatory state termed ‘inflammaging’, which contributes to the development of several age-related diseases [53]. The impact of ageing has been studied in numerous experimental disease models that collectively show a general increase in inflammation, organ damage and enhanced mortality [54]. These events are often associated with increased neutrophil tissue infiltration [55,56]. Despite these findings, the impact of increased age on circulating neutrophil numbers is unclear, with mixed reports in the elderly and in aged experimental mammals [57]. In humans, increased leucocyte blood counts, most notably neutrophils, have been directly related to the endothelium dysfunction associated with ageing and growing susceptibility to cardiovascular disease [58]. Findings related to altered functions of neutrophils from aged individuals are similarly inconsistent, though in general there appears to be an impairment of phagocytosis and bacterial killing, as well as reports on impaired intracellular signalling pathways [57,59,60]. Several studies have also indicated broad differences in surface receptor expression on neutrophils from older individuals, collectively suggesting alterations in adhesive capacity, responsiveness or intracellular signalling [57,59–61]. For example, there is evidence for increased levels of CD18 and reduced levels of ICAM-3 in human neutrophils in the elderly [62,63], and reduced CXCR2 signalling in neutrophils from aged mice [64]. In contrast, the expression of several other key surface receptors is reportedly unchanged in neutrophils from aged individuals, including toll-like receptor 2 (TLR2), TLR4 and granulocyte-macrophage colony-stimulating factor (GM-CSF) receptor [61]. Collectively, with increasingly diverse expression changes reported, at present there are no definitive cell surface markers for neutrophils from older individuals.

Clearer data indicate transcriptional differences in neutrophils from aged donors, when compared with neutrophils from young donors. Several of these changes are not only in the expression of surface molecules, but also include cytokine production machinery, such as reduced mRNA for IL-1β-converting enzyme [65]. These findings also follow through to protein expression levels, where neutrophils from aged mice show differential expression of several cytokines at rest and also following stimulation, which may contribute to their functional defects [66]. Multiple studies have also suggested that age-related functional defects may be attributed to decreased intracellular signalling efficiency, including changes in membrane fluidity, lipid raft functioning, basal calcium levels and in regulation of the actin cytoskeleton [61]. Such changes have the potential to present as wide-ranging functional defects, possibly occurring in a stimulus-specific manner or downstream of receptors that show little age-related change in expression. There are also indications of reduced apoptosis of neutrophils isolated from aged donors. For example, Gasparoto *et al.* [67] recently described neutrophils from elderly human donors to persist longer in culture without stimulation, and a lower rate of spontaneous apoptosis was noted in a separate study with a similar population of subjects [62]. However, the above findings are in conflict with reports of neutrophils from aged individuals being more susceptible to inflammatory-induced apoptosis and unable to be rescued by cytokines such as GM-CSF, unlike their counterparts in younger individuals [68]. These results seem independent of inflammatory neutrophilia, which appears to occur to a similar magnitude in elderly and younger subjects [60]. In terms of recruitment into tissues, leucocytes from aged donors show increased rolling [69], though the impact of age on neutrophil firm adhesion and transmigration is less clear.

Together, these studies suggest the occurrence of complex phenotypic changes in neutrophils of aged individuals, although, owing to confounding factors, such as the increased number of medications taken by the elderly population or differences in monitoring protocols, it has proved difficult to obtain consistent and reliable information on age-related neutrophil phenotype changes. Another key variable may well be the impact of senescent vascular components (e.g. endothelial cells) on neutrophil activation/priming status, a phenomenon that may also impact neutrophil phenotype and responses in the elderly. Collectively, while it appears that several neutrophil states occur during the ageing process of the host, without doubt, the occurrence, extent and transition between different states is highly complex, and likely to be modulated by numerous exogenous and endogenous factors.

## 4. Pathologically induced changes in neutrophil phenotypes

In addition to phenotypic changes in neutrophil function under homeostatic and physiological conditions, numerous changes in neutrophil phenotype and their potential functional consequences have also been recognized in the context of pathological conditions (table 2).

**Table 2.** Neutrophil subsets during pathological conditions. The table describes neutrophil subsets, including their phenotype and function, which have been identified in pathological conditions such as cancer, infection and inflammation in mice or humans. Subsets with a pro-inflammatory or anti-inflammatory phenotype are highlighted in italics and bold, respectively. IFN, interferon; IL, interleukin; i.v., intravenous; LDG, low-density granulocyte; LPS, lipopolysaccharide; MRSA, methicillin-resistant *Staphylococcus aureus*; PBMC, peripheral blood mononuclear cell; PMN, polymorphonuclear neutrophil; ROS, reactive oxygen species; SIRS, systemic inflammatory response syndrome; SLE, systemic lupus erythematosus; TAN, tumour-associated neutrophil; TGF, transforming growth factor; TLR, toll-like receptor.

disease model	subset	occurrence	phenotype	proposed systemic effects
murine subcutaneous tumours [10]	*N1 TAN*	— *tumours with TGF-*β* inhibition*	— *hypersegmented nuclear morphology* — *high ROS production* — *high Fas, ICAM-1 and TNF-*α* expression* — *higher tumour cell cytotoxicity* in vitro	— *pro-inflammatory and anti-tumorigenic*
	**N2 TAN**	— **majority of untreated tumours**	— **normal, segmented nuclear morphology** — **suppress T-cell effector functions via arginase**	— **immunosuppressive and pro-tumorigenic**
MRSA infection in mice [11]	*PMN-I*	— *infected mice with mild SIRS*	— *normal, segmented nuclear morphology* — *induce classically activated macrophages* — *produce IL-12 and CCL3* — *express TLR-2, -4, -5 and -8 and integrin *α*4*	— *protection from MRSA infection*
	**PMN-II**	— **infected mice with severe SIRS**	— **ring-shaped nuclear morphology** — **induce alternatively activated macrophages** — **produce IL-10 and CCL2** — **express TRL-2, -4, -7 and -9 and integrin αM**	— **susceptibility to MRSA infection**
human experimental endotoxemia [12,14]	**CD16^dim^/CD62L^bright^**	— **post-LPS i.v.; up to 50% of neutrophils**	— **immature, banded nuclear morphology** — **lower ROS production and interaction with opsonized bacteria** — **increased survival *in vitro***	— **increased susceptibility to infection during SIRS**
	**CD16^bright^/CD62L^dim^**	— **post-LPS i.v. or trauma; 10–15% of neutrophils**	— **hypersegmented nuclear morphology** — **increased capability to produce ROS** — **express integrins αM and αX and ICAM-1** — **integrin αM and ROS-dependent inhibition of T-cell proliferation**	— **immunosuppressive; suppress T-cell proliferation**
	*CD16^bright^/CD62L^bright^*	— *normally occurring neutrophils*	— *normal, segmented nuclear morphology* — *higher ROS production and interaction with opsonized bacteria* — *express integrin *α*M and Fc*γ*RII*	— *excessive tissue damage during SIRS*
SLE patients [13,70]	*LDG*	— *about 17% of PBMCs in SLE patients*	— *immature, banded/lobular nuclear morphology* — *activated phenotype: express CD66b, integrin *α*M*β*2, type I interferons, TNF-*α* and IFN-*γ** — *reduced phagocytic ability* — *increased capacity to form NETs* — *higher endothelial cell cytotoxicity* in vitro	— *pro-inflammatory*

### 4.1. Cancer

Emerging evidence suggests that cancer is associated with the presence of specialized neutrophil subsets [71]. Analysis of multiple cancer models has demonstrated that a sizeable portion of the immune cell infiltrate consists of tumour-associated neutrophils, termed TANs. It is now clear that TANs are a distinct cell population with a unique transcriptional profile when compared with naive bone marrow neutrophils [72]. Furthermore, tumour-associated neutrophils can have considerable plasticity in that to date two subsets of TANs with distinct phenotypes and properties have been characterized [10,73]. Briefly, the majority of TANs exhibit an immunosuppressive and pro-tumorigenic phenotype, a subset termed N2. Interventions such as blockade of transforming growth factor-β lead to the formation of an immunostimulatory and anti-tumour phenotype TAN subset termed N1. These terminologies are in line with the well-established tumour-associated macrophage subsets, the pro-tumorigenic M2 or the anti-tumorigenic M1 subsets. N1 neutrophils are characterized by increased expression levels of Fas, immunostimulatory cytokines and, chemokines, and, interestingly, increased cell surface expression of ICAM-1. In addition, they exhibit stronger cytotoxicity towards tumour cells *in vitro* and are characterized by a hypersegmented and lobulated nuclear morphology [10]. Melanoma patients also show a specialized subset of immunosuppressive neutrophils in their blood that is induced by serum amyloid A1 (SAA-1), produces the anti-inflammatory cytokine IL-10 and is able to suppress tumour-specific CD8^+^ T-lymphocyte proliferation *in vitro.* Intriguingly, SAA-1 also promotes the interaction of these neutrophils with invariant natural killer T cells that decreases their IL-10 production while enhancing IL-12, thereby dampening their immunosuppressive properties, again resulting in the existence of two distinct neutrophil populations with defined functions [74].

### 4.2. Infection

Infections with methicillin-resistant *Staphylococcus aureus* (MRSA) are a serious problem in patients who have undergone traumas such as severe burn or major surgery. Neutrophils, as innate immune effector cells, play an important role in the clearance of MRSA. Interestingly, two different neutrophil (PMN) subsets have been identified in a murine model of MRSA infection (PMN-I and PMN-II) [11]. These subsets develop from normal neutrophils (PMN-N) in response to different circumstances within the infected mouse. PMN-N in MRSA-infected mice suffering from mild systemic inflammatory response syndrome (SIRS; induced by lightly scalded burn injuries) develop into PMN-I. MRSA infection in mice with severe SIRS (induced by severely flamed burn injuries) leads to the formation of PMN-II neutrophils. The two subsets differ with respect to their expression of cytokines and chemokines, TLRs and adhesion molecules, as detailed in table 2. These unique expression profiles lead to the induction of either classically or alternatively activated macrophages by IL-12-expressing PMN-I or IL-10-expressing PMN-II, respectively. Because classically activated macrophages are much more efficient anti-bacterial effector cells, PMN-I neutrophil induction results in the clearance of MRSA infection, whereas animals with PMN-II neutrophils succumb to the bacterial challenge. These findings demonstrate that the noted changes in neutrophil phenotype are of vital importance to the survival of the host organism [11]. Neutrophils resembling the PMN-I and PMN-II subsets have also been detected in a mouse model of *Candida albicans* infection. A CD80^+^ subset of neutrophils that was expanded upon exposure to *Candida* hyphae expressed IL-10 and inhibited proliferation of CD4^+^ T cells [75]. Further evidence for the presence of an IL-10-expressing neutrophil subset with immunosuppressive functions comes from a study of *Trypansoma cruzi* infection in mice. In this model, the induced IL-10-producing neutrophils were recruited to the liver in an IL-17RA-mediated fashion, where they not only helped to destroy parasites, but also limited excessive tissue damage by inhibiting T-cell proliferation and interferon-γ (IFN-γ) production [76]. In addition to bacterial infection models, specialized subsets of neutrophils have also been described within a model of viral infection. Specifically, a distinct population of neutrophils with a CD11b^+^Ly6C^+^Ly6G^+^ phenotype was noted in vaccinia virus-infected tissues. These specialized neutrophils produce high levels of type I IFNs and contribute to the protection of virus-infected organs from excessive immune-mediated tissue damage via a mechanism involving the ability of these cells to generate high levels of ROS [77].

### 4.3. Sepsis

More than 25 years ago, human neutrophils were reported to show heterogeneity with regard to the expression of CD16, an Fc receptor that induces oxidative burst and phagocytosis upon immunoglobulin G binding [78,79], but only recently have the underlying mechanisms and functional consequences of this heterogeneity been addressed. In severe systemic inflammation elicited by trauma or sepsis, neutrophils play a paradoxical role. They contribute to collateral tissue damage during the initial inflammatory SIRS phase of sepsis. However, most deaths occur during the later compensatory anti-inflammatory response syndrome stages of the disease, when patients develop immune suppression and succumb to additional infections [80]. The latter indicates that despite notable neutrophilia, the host is immunocompromised and more prone to infections [81], suggesting alterations in the effector functions of neutrophils. An elegant explanation for this seemingly paradoxical situation has been offered by Pillay and co-workers using a human model of endotoxemia [14,82]. The authors report that 3–6 h post-administration of a systemic low dose of lipopolysaccharide, there is a marked change in the profile of circulating neutrophils leading to the presence of a functionally heterogeneous neutrophil pool. Within this, newly released CD16^dim^ neutrophils were found to have reduced capability to interact with opsonized bacteria and generate ROS, while differentiated CD16^bright^ neutrophils showed enhanced anti-microbial function and ROS generation [14]. Based on these results, it is hypothesized that in sepsis the CD16^dim^ neutrophil subset may account for increased susceptibility to infections, while the activated CD16^bright^ neutrophils may contribute to tissue damage. In a subsequent study, the authors show that systemic LPS leads to the presence of a previously undescribed neutrophil subset characterized by a distinct CD16^bright^CD62L^dim^CD11b^bright^CD11c^bright^ phenotype that was also detected in patients who had suffered severe injury [12]. This neutrophil subset exhibited suppressive effects on T-cell proliferation, a response that was mediated by neutrophil expression of Mac-1 and locally released ROS into the immunological synapse between neutrophils and T cells. Further characterization of these immunosuppressive neutrophils identified them as exhibiting a hypersegmented nuclear morphology, indicative of the cells being mature, as well as expressing high levels of ICAM-1. As the latter is a key characteristic of neutrophils that have undergone rTEM [16,17], these findings raise the intriguing possibility that tissue-infiltrated neutrophils returning back into the circulation may contribute to the noted CD16^bright^CD62L^dim^ neutrophil population. Overall, the above findings have identified a novel subset of neutrophils that are not part of the myeloid-derived suppressor cells (MDSC). MDSC are a leucocyte subset described in pathologies such as cancer, infection and inflammation that contains mainly immature granulocytic and monocytic cells, and is capable of suppressing T-cell proliferation mainly via arginase I and ROS-dependent mechanisms [83].

### 4.4. Systemic lupus erythematosus

Systemic lupus erythematosus (SLE) is an autoimmune disease directed against nuclear antigens, and is characterized by pro-inflammatory immune complex deposition and widespread tissue inflammation. Recently, SLE has been associated with a specific neutrophil subset [84]. The blood of SLE patients contains a pro-inflammatory neutrophil subset termed low-density granulocytes (LDG), identified owing to their localization in the peripheral blood mononuclear cell (PBMC) fraction during density gradient sedimentation. These cells express higher levels of type I IFNs, TNF and IFN-γ, and show reduced phagocytic capacity when compared with normal neutrophils from both lupus patients and healthy controls [13]. LDGs account for around 17 per cent of PBMCs in blood from SLE patients [13]. Furthermore, new evidence shows that NETosis, neutrophil cell death via the formation of NETs, plays an important role in lupus pathogenesis [84]. An elegant study linking these two phenomena has demonstrated that LDGs spontaneously undergo NETosis, offering an explanation for the pathogenic potential of this neutrophil subset. Of relevance, enhanced NET formation increases the number of circulating NET-derived immune complexes that can stimulate plasmacytoid dendritic cells to release IFN-α, which in turn further enhances NET formation in normal lupus neutrophils, causing a positive feedback loop of NET release and its associated tissue-damaging effects [70].

## 5. Conclusion

It is becoming increasingly evident that far from being a homogeneous cell population, neutrophils display a vast degree of plasticity and heterogeneity within a wide range of physiological and pathological scenarios. This shows stark parallels with our current understanding of lymphocyte and monocyte heterogeneity, and suggests that future discussions and investigations of immune cell subsets should be highly inclusive of neutrophils. Of particular importance, while there is a growing body of evidence for the existence of certain neutrophil subsets in disease conditions, for example as identified in cancer, sepsis, trauma and ischaemia–reperfusion injury, there is a need for greater understanding of their characteristics, prevalence and pathogenic potential. More insight into this developing aspect of neutrophil biology could pave the way to successful and selective targeting of pathogenic neutrophil subsets, a novel therapeutic strategy that may be beneficial for treatment of inflammatory conditions without compromising the innate immune response.

## 6. Acknowledgements

The work in our laboratory is largely funded by the Wellcome Trust (Senior Investigator Award to S.N., ref: 098291/Z/12/Z). M.B. is supported by a Marie Curie Fellowship from the European Union.
